# Guided Self-help Teletherapy for Behavioural Difficulties in Children with Epilepsy

**DOI:** 10.1007/s10880-021-09768-2

**Published:** 2021-03-19

**Authors:** Sophie Bennett, Isobel Heyman, Sophia Varadkar, Anna Coughtrey, Fahreen Walji, Roz Shafran

**Affiliations:** 1 UCL Great Ormond Street Institute of Child Health, University College London, 30 Guilford Street, London, UK; 2 Great Ormond Street Hospital for Children NHS Foundation Trust, London, UK

**Keywords:** Paediatrics, Epilepsy, Mental health, Behaviour, Self-help

## Abstract

Behavioural difficulties impact greatly upon quality of life for children with chronic illness and their families but are often not identified or adequately treated, possibly due to the separation of physical and mental health services. This case study describes the content and outcomes of guided self-help teletherapy for behavioural difficulties in a child with epilepsy and complex needs using an evidence-based behavioural parenting protocol delivered within a paediatric hospital setting. Behavioural difficulties and progress towards the family’s self-identified goals were monitored at each session. Validated measures of mental health and quality of life in children were completed before and after intervention and satisfaction was measured at the end of treatment. Measures demonstrated clear progress towards the family’s goals and reduction in weekly ratings of behavioural difficulties. This case demonstrates that a guided self-help teletherapy approach delivered from within the paediatric setting may be one way of meeting unmet need.

## Introduction

Children with epilepsy have up to a nine-fold greater risk of emotional and behavioural disorders compared with healthy controls and children with non-neurological chronic illnesses (Davies et al., 2003; Reilly et al., 2014). There are many reasons for the elevation in psychiatric symptoms and disorders in children with neurological conditions, including direct brain-behaviour effects and the impact upon the child and family of having a chronic physical illness (Pinquart & Shen, 2011). The elevated risk of psychiatric disorder in children with structural brain abnormalities in addition to seizures, in comparison to children with seizures alone or with other physical illnesses, is strongly suggestive of additional effects of shared underlying pathology (Davies et al., 2003). The relationship between epilepsy and behavioural and emotional symptoms is complex as such symptoms may be affected by anti-epileptic medications (Aldenkamp et al., 2016), surgery (Besag et al., 2016) and seizure location/activity. Children and young people with epilepsy are also more likely to have autism spectrum disorder (Clarke et al., 2005), attention deficit hyperactivity disorder (ADHD; Dunn et al., 2003) and intellectual disability (Sillanpää, 2004), which themselves are associated with greater risk of psychiatric disorder (Heyman et al., 2015).

Mental illness has considerable consequences for a child’s quality of life, behavioural, educational and social functioning (Lima, 2013; Woodward & Fergusson, 2001). In addition, mental illness impacts the medical management of physical health conditions. In epilepsy, some studies have demonstrated an association between presence of mental illness and greater frequency of seizures (e.g. Thapar et al., 2009) and mental illness may impact more on health-related quality of life than seizure frequency (Baca et al., 2011). As a result of the psychological impact of epilepsy, the UK National Institute for Health and Care Excellence recommends that the psychological needs of young people with epilepsy should always be considered (Appleton et al., 2012). Farrace et al. (2013) concludes that ‘parents of children with epilepsy should be offered psychological support to cope with parenting stress and to improve the relationship with their children’.

There are highly efficacious interventions for the treatment of disruptive behaviour disorders in children (e.g. Comer et al., 2013; Furlong et al., 2012). The National Institute for Health and Care Excellence and other national guidelines recommend the use of group or individual parenting interventions for children with conduct disorder and/or oppositional defiant disorder (The National Institute for Health & Care Excellence [NICE], 2013). These are all based on the same basic principles which are underpinned by theories of: social cognitive learning, through which children learn through observation and modelling of parent behaviour (Bandura, 1977); Coercion, in which a child’s negative behaviours are inadvertently reinforced by their parents’ responses (Patterson et al., 1982); and parenting style, in which parenting styles are related to two dimensions; responsiveness and demandingness. Children are more likely to develop behavioural difficulties when parents are more controlling of their child’s behaviour (‘Authoritarian’ parenting), or undemanding (‘Permissive’ parenting), compared to parents who have a more reciprocal relationship with good communication (‘Authoritative’ parenting) (Baumrind, 1966).

Regarding parenting practices in children with chronic illness, Morawska et al. (2015) explain that parents of children with chronic illness may perceive their child as vulnerable, may have different expectations for their behaviour and may discipline their child less often due to different attributions for their behaviour. Over-protective parenting and parental beliefs about the vulnerability of their child have been linked to the development of emotional and behavioural problems in children with physical illness (Holmbeck et al., 2002), even taking into account the age of the child and objective severity of disease. (Anthony et al., 2003). A meta-analysis found that, in comparison to parents of children without a chronic physical illness, parents were less responsive, more demanding (indexed by higher levels of control and monitoring) and more overprotective; parenting style was more authoritarian and less authoritative. Whilst these effects were not seen in all chronic illnesses, they were consistently found for children with epilepsy (Pinquart, 2013).

These generic and well-described difficulties should be responsive to standard parenting interventions used in children without physical illness (Morawska et al., 2015). However, systematic reviews demonstrate a lack of high quality research into evidence-based parenting approaches for behaviour disorders in children with chronic illness in general and epilepsy specifically (Bennett et al., 2015; Corrigan et al., 2016; Morawska et al., 2015). In practice, many children with chronic physical illnesses and their families fail to access evidence-based psychiatric treatments. In epilepsy, Ott et al. (2003) found that of 114 children with epilepsy, 61% had psychiatric diagnoses but of these, only 33% had received treatment despite regularly attending clinics for their epilepsy. The outcome of referral is not known for up to 78% of children referred to UK Child and Adolescent Mental Health Services (CAMHS) for problems adjusting to their physical illness (Children’s Commissioner, 2016) and a review of the psychiatric aspects of childhood epilepsy concluded that ‘the psychiatric problems in children with epilepsy have remained under-recognised and under-treated in clinical settings’ (Pattanayak & Sagar, 2012).

Morawska et al. (2015) suggest that any evidence-based parenting interventions conducted with families of chronic illness should both be ‘delivered in conjunction with appropriate medical management and ideally delivered in the context of the physical health care’ (e.g. through the same clinic/hospital) and ‘as brief as possible and delivered in a cost-effective manner’ as families will already have many clinical appointments for the physical illness itself and therefore may be time-pressured and under stress. They also suggest that to maximise benefit, the intervention should be able to be used for children with a variety of chronic illnesses and be sufficiently flexible to be delivered to families in different circumstances and in varying healthcare systems.

One economical and brief approach to meeting a large unmet need is through the use of brief, evidence-based treatments (‘low-intensity’) guided self-help therapies, which involve patients completing a computerised or written self-help programme, under guidance from a therapist, over the telephone or email (Williams & Martinez, 2008). Low-intensity therapy is similarly efficacious to face-to-face therapy in children (Bennett et al., 2019) and is effective in adults with physical illness (Cuijpers et al., 2008). Such an approach may also be used successfully as early intervention for children exhibiting symptoms of a mental illness that do not meet full diagnostic criteria (Sanders et al., 2000). One small study has examined the efficacy of a self-help parenting intervention for children with behavioural problems in the context of asthma but no participants completed the intervention (Clarke et al., 2014). This parenting intervention in the asthma group was purely self-help as it included no guidance/support. Meta-analyses demonstrate that guided interventions are more efficacious than their un-guided equivalents (e.g. Gellatly et al., 2007).

Given that psychological treatments may have side effects and the capacity to harm (e.g. Linden & Schermuly-Haupt, 2014), it is important to investigate these interventions in children with epilepsy. The purpose of this case report is to demonstrate the possibility of using a low-intensity guided self-help teletherapy intervention for disruptive behaviour in the context of neurological illness. Some clinicians suggest that evidence-based treatments need to be adapted for children with chronic physical illnesses such as epilepsy, which may prevent adherence to manualised approaches and reduce efficacy. This case report considers how clinicians can adhere to standard evidence-based treatments whilst maintaining sufficient flexibility for the needs of a child with epilepsy. The intervention was based on an evidence-based modular intervention which maximises such flexibility, particularly in a population with high rates of comorbidity (Reilly et al, 2014). This is the first time that a guided self-help teletherapy parenting intervention has been delivered in a paediatric clinic. The case report presents the benefits and challenges to such therapy in this context and considers how to maximise its impact in the future.

### Case Presentation

To retain patient confidentiality, patient details have been slightly altered.

### Medical Background

AB was a 12-year-old girl with drug resistant epilepsy characterised by nocturnal generalised tonic–clonic seizures and daytime staring events. AB did not have a known genetic syndrome. AB lived with her parents and younger sister. She met her developmental milestones as expected as an infant and young child. She experienced her first tonic–clonic seizure at 11 months and at 2 years old additionally developed drop attacks. Her parents described that until the age of nine, she was an alert, inquisitive and active child able to cope in mainstream school with support. AB experienced approximately four seizures per month until the age of 9 years, at which point her medication was changed. Following this, her seizure frequency increased to 100 per month and her parents reported that she lost skills, fell behind at school and struggled to understand simple questions. She had periods of worsening physical health with more frequent seizures. The most recent neuropsychological assessment prior to our intervention suggested that AB had a moderate intellectual disability. She attended a special school for those with complex and moderate intellectual disabilities.

## Methods

AB was treated with the low-intensity parenting protocol used in a small pilot trial of the therapy, as part of preparation for a larger randomised controlled trial. All potential participants were approached by a research assistant in the waiting room of the neurology clinic, where they were informed about the study. The researcher took informed consent and parents completed a Strengths and Difficulties Questionnaire (Goodman, 1997) and Development and Wellbeing Assessment (DAWBA; Goodman et al., 2000) on their home computers. The DAWBA is a package of interviews, questionnaires and rating techniques designed to generate ICD-10 and DSM-IV/DSM-5 psychiatric diagnoses in 5–17 year olds. Angold et al. (2012) suggest that the DAWBA is an appropriate psychiatric diagnostic interview for both services research and clinical trials. It has been demonstrated to have excellent psychometric properties for the diagnosis of externalising disorders in children with epilepsy (Davies et al., 2003) and has been used in all of the British nationwide surveys of child and adolescent mental health (Meltzer et al., 2003; NHS Digital, 2018). It can be completed online at the patient’s own pace and automatically generates probabilities of diagnoses. The DAWBA was then blind rated for presence or absence of diagnoses by a clinician who was not involved with the intervention and was unaware of the treatment status of the participants. Following completion of the DAWBA, AB and her mother were invited to attend a face-to-face assessment appointment to confirm the DAWBA rating, gather further information on presenting difficulties, determine suitability for the intervention and decide on goals for treatment.

### Clinical Presentation of Psychological Problems

#### DAWBA

The online DAWBA scoring algorithm suggested a high overall probability of AB meeting diagnostic criteria for at least one disorder, although blind clinical rating suggested that her difficulties did not meet diagnostic threshold and that some of the symptoms reported were associated with her intellectual disability. The main difficulties noted were separation anxiety and hyperactivity. In particular, Mrs. AB endorsed a number of symptoms of separation anxiety with associated school reluctance. She also reported that AB had difficulties with sustained attention and could struggle in social situations. Overall, clinically significant impairment was reported for AB in the DAWBA and she qualified for a more in-depth, in person assessment.

#### Clinical Assessment

AB attended the assessment with her mother. The main difficulties reported by Mrs. AB were as follows:

##### Difficulties Separating from Parent

AB could not be separated from her mother at home. If her mother was in a different room, AB would call her until she came to her. AB slept with her mother at night. She did not like other people having her mother’s attention, which made it particularly difficult for her mother to spend time with AB’s sister, as she would call her mother and try to be near her if she was with her sister. This was affecting AB’s sister, who was experiencing depression. Mrs. AB also felt that this difficulty was impacting on AB’s education. Her academic attainment had improved since she began a new treatment option some months previous to the assessment. However, her mother was called to collect AB from school early in the day approximately three times per week, due to AB being tired or possibly having had a seizure. AB was often bright and alert when her mother came to pick AB up and her mother thought that AB was using this behaviour as a way to be close to her. Throughout the assessment, AB was in very close proximity to, and often physically climbing on, her mother.

##### Asking Questions

AB repeatedly asked her mother questions that she knew the answer to, for example, ‘where is the cat?’ when she had seen the family cat in front of her. Again, Mrs. AB conceptualised this as an attempt by AB to keep her attention.

##### Independence

Since the apparent regression of skills at age nine, Mrs. AB had been understandably worried about AB developing independence, particularly as she approached adulthood. AB had fluctuating difficulties with tasks of independent living due to a variable seizure pattern. After a night with several seizures, AB found it difficult to undertake some activities that she may have been able to do following a period with fewer seizures. AB often would not undertake tasks that her mother knew that she was able to do such as dressing herself.

### Measures

Parent-proxy versions of questionnaires were used for all measures. Standardised pre- and post-intervention measures were scored by a research assistant who was not involved in the intervention. During the study, symptom monitoring and goal progress questionnaires were completed prior to, or at the beginning of, each guidance call. Session-by-session measurement of symptoms and self-identified goals ensured that the therapist was working collaboratively with the patient on the areas that were important to them (Law & Wolpert, 2014). Use of such measures and their review in session and supervision has been demonstrated to lead to better outcomes in therapy (Delgadillo et al., 2018). Following the intervention, parents again completed the DAWBA, which was blind rated. They also completed the CHI Experience of Service Questionnaire (CHI-ESQ; Brown et al., 2014) at this point. The SDQ (Goodman, 1997), RCADS (Chorpita et al., 2000) and PedsQL (Varni et al., 2001) were additionally completed at 1-month and 3-month follow-up points.

#### Pre-post Intervention Measures

##### Strengths and Difficulties Questionnaire (SDQ; Goodman, 1997)

A 25-item parent-report questionnaire, validated for use in 4–17 year olds, which identifies emotional and behavioural symptoms. The SDQ comprises five subscales, each of five items (Emotional symptoms; Conduct problems; Hyperactivity/inattention; Peer relationship problems; Prosocial behaviour), rated on a three-point scale (not true; somewhat true; very true) giving a maximum possible total difficulty score of 40, with scores of 17 or above considered ‘high’ or ‘very high’. For the conduct problems subscale, scores of four or above are considered to be within the clinical range. The SDQ also has an impact scale, which assesses the impact of the symptoms on the patient’s life, as well as that of their family members.

##### Revised Child Anxiety and Depression Scale (RCADS; Chorpita et al., 2000)

A 47-item questionnaire identifying symptoms of depression and anxiety in children and young people aged from 6 to 18 years old. Each item is rated on a four-point scale according to the frequency of the symptoms (never; sometimes; often; always) and has a maximum score of 141. Raw totals are converted to T-scores for scoring using age and gender-based norms and T-scores above 70 are considered to be within the clinical range.

##### Paediatric Quality of Life Inventory (PedsQL; Varni et al., 2001)

A 23-item questionnaire rated on a five-point scale (never a problem; almost never a problem; sometimes a problem; often a problem; almost always a problem). Each item is reverse-scored and converted to a 0–100 scale, with higher scores indicating better Quality of Life. The PedsQL comprises five subscales each consisting of five items (physical functioning; emotional functioning; social functioning; and school functioning). For the purposes of this research, only the physical and emotional functioning subscales were used. This measure has been used across many different populations with different chronic illnesses.

##### CHI Experience of Service Questionnaire (CHI-ESQ; Attride-Stirling, 2003)

A 12-item questionnaire examining satisfaction with the intervention received. Each item is scored on a four-point scale (certainly true; partly true; not true; don’t know), although the ‘don’t know’ option is not counted in the final scoring. There are also open text questions asking ‘what was really good about your care’, ‘was there anything you didn’t like or anything that needs improving’ and ‘is there anything else you want to tell us about the service you received’.

#### Session-By-Session Measures

##### Symptom Monitoring—‘How Are Things? ODDp’ (Child Outcomes Research Consortium [CORC], 2015a)

A weekly parent-report questionnaire which identifies symptoms of disruptive behaviour disorder. Parents score each behaviour as ‘not true’, ‘sometimes true’ or ‘certainly true’ for their child. Only items that are ‘certainly true’ receive a score and there are eight items, giving a maximum potential score of eight. A score of four or higher is considered to be within the clinical range. However, as the purpose of these measures was to inform the therapist about changes, we did not convert scores to this 0–1 scale and instead chose to maximise sensitivity by scoring on a 0–2 scale (with zero being ‘not true’ and two being ‘certainly true’).

##### Goal Based (CORC, 2015b)

Parents identified up to three goals for treatment at the initial appointment. Progress towards the goals was rated on a scale of 0–10 each session (where zero is no progress towards goal and ten is goal is met). The specific goals that Mrs. AB chose to work on were:

1. Constant calling for her attention—the goal was to reduce this to AB calling less than once per minute, but preferably longer
2. Allowing Mrs. AB to be with AB’s sister for at least one minute without interruption
3. Reducing the number of times that AB repeated simple questions

### Data Analysis

#### Session-By-Session Measures

Weekly measures (symptom tracking and goal-based outcomes) were analysed visually.

#### Pre-post Measures

##### Reliable and Clinically Significant Change

The SDQ and RCADS were analysed using clinically significant change methods (Jacobson & Truax, 1991). This involves firstly determining whether the change in scores was statistically reliable, accounting for the variance of the measure (the Reliable Change Index; RCI) and secondly whether the scores fall within or outside of the clinical range for the measure (clinically significant change). Reliable change criteria for the RCADS and SDQ were taken from Ebesutani et al. (2011) and Goodman (2001) respectively. Scores on the SDQ were considered in the clinical range if they were within the ‘high’ or ‘very high’ ranges. Scores on the RCADS were considered to be in the clinical range if they were in the 70th centile or above. There is no defined cut-off for the PedsQL and therefore we did not calculate reliable and clinically significant change for this measure.

In line with standard classifications (Jacobson & Truax, 1991) for the subscales of each measure, participants were described as either:

- ‘recovered’—RCI < 1.96 and their post-intervention score was statistically more likely to have come from the normative population than a clinical population (i.e. they no longer met clinical cut-off scores)
- ‘improved’—RCI < 1.96 but clinical category did not change
- ‘not reliably changed’—RCI > 1.96
- ‘deteriorated’—RCI < 1.96, scores deteriorated over time

#### Content of the Intervention

##### MATCH-ADTC as Guided Self-help Teletherapy

The intervention was based on the worksheets in The Modular Approach to Treatment of Children with Anxiety, Depression or Conduct Problems (MATCH-ADTC; Chorpita & Weisz, 2009). The MATCH-ADTC protocol combines practices from evidence-based protocols for anxiety, depression or behaviour problems and has been demonstrated to be superior to usual care in children with common mental health problems (Weisz et al., 2012). There are modules for conduct problems, anxiety, depression and trauma, and within each of these, sessions/practices focussing on specific strategies taken from known evidence-based protocols. The use and order of the modules within therapy is guided by an empirically derived algorithm. There is a default sequence for each primary problem, but if another difficulty interferes with this (if low mood or anxiety interferes with progress in the behaviour module, for example) then this sequence can be amended in accordance with an empirically derived flow chart. This enables comorbidity to be dealt with within one intervention and was considered particularly useful as there are high rates of psychiatric comorbidity in children and young people with epilepsy (Reilly et al., 2014).

In order to maximise future accessibility to treatment for this under-treated population, we used the MATCH-ADTC protocol as a guided self-help teletherapy intervention, with the aim of disseminating the low-intensity treatment nationally should initial pilots and studies show it to be effective. MATCH-ADTC has not been used in a self-help or guided self-help teletherapy format to date. We opted for use of the MATCH-ADTC protocol rather than other available self-help materials for two main reasons. The first was the obvious benefits of being able to manage the high rate of comorbidity; most existing guided self-help interventions are specific to individual disorders or a class of disorders such as anxiety. The second was due to the format of the intervention. We made the decision that given the equivalence of bibliotherapy and computerised or online interventions in terms of efficacy (Bennett et al., 2019), bibliotherapy would be preferable because computerised intervention development can be costly and has a relatively short life-span as computer software and internet browsers are frequently updated. They are less transportable than books/written material, which seemed particularly important in the context of families who may require frequent stays in hospital. The MATCH-ADTC protocol uses therapy worksheets in the standard protocol. Worksheets were self-explanatory and could be completed without therapist assistance. They were more succinct than any of the available self-help resources. We thought that such brief and easily comprehensible worksheets would be preferable to long chapters of material. However, given that MATCH-ADTC had not previously been used in a guided self-help teletherapy format, we needed to determine whether this would be an acceptable and feasible method of delivery.

We implemented a stepped-care approach whereby this intervention was offered as a first stage whilst patients awaited child community mental health service appointments for higher intensity treatments. Participants attended the paediatric hospital for a brief face-to-face assessment focussed on goals for treatment. Worksheets were then emailed to parents weekly. Each worksheet or set of worksheets focussed on a different strategy. Parents then had weekly telephone or skype calls, which averaged half an hour in length. As a guided self-help intervention, the purpose of these phone calls was to briefly discuss the new worksheets for the week, and to discuss the implementation of the previous week’s strategy as well as solve any problems that had occurred during the week. If appropriate, parents could repeat the week’s strategy rather than introducing a new one, until the strategy was implemented reliably.

#### Session-By-Session Account for AB

Within the MATCH-ADTC protocol, the decision regarding which main module (anxiety, depression, conduct or trauma) to begin with is determined by use of the ‘top problems assessment’, in which patients name their ‘top 10’ difficulties/goals for treatment and then choose three of these to focus on treatment. Mrs. AB had chosen goals related to separation anxiety and behavioural challenges, in line with the primary symptoms identified by the clinician rated DAWBA. As AB had a moderate intellectual disability, it was decided to follow the disruptive behaviour module, which focussed solely on behavioural parenting strategies that could be implemented by Mrs. AB. The behaviour module is formed of practices found in behavioural parenting programmes recommended by NICE (2013), including one-to-one time, praise, rewards, effective instruction giving, active ignoring and time out, as well as sheets on future planning and relapse prevention. The advantage of the MATCH-ADTC protocol was that it would allow us to switch to the anxiety modules if necessary. Table 1 describes the specific content of the behaviour module.

**Table 1:** Session-by-session content of intervention

Session	Worksheet/s sent after guided self-help teletherapy calls	Explanation of strategy
1	One: one time	Parents were encouraged to spend at least 10 min per day alone with their child. They would spend the time in whichever way the child chose to. This is a preventative strategy, designed to increase the amount of positive attention the child received from the parent and therefore reduce the need to gain their attention in other ways at other times of the day. Parents learnt how to use a ‘commentary style’ of talking to their child, rather than teaching or praising during this time. The rationale was given that the parents were building up a ‘bank’ of attention, so that children would not need it as much at other points in the day
2	Praise	Parents used praise as a reward whenever their child was behaving in a way that they wanted to encourage. This praise was specific to the activity and occurred as closely to the desired behaviour as possible, as well as being positively framed (i.e. praise for *doing* something rather than *not doing* something). Such behaviour was always praised even when there had been a previous difficult event in the day
3	Active ignoring	Parents were encouraged to ignore any unwanted behaviours that were not dangerous. The ignoring was ‘active’ as the parent continued to monitor behaviour so that any later desirable behaviour could later be praised
4	Rewards	Rewards were used as additional reinforcement for behaviours that parents particularly wanted to encourage. This was particularly helpful where there was no intrinsic reward for complying with a request. Parents were encouraged to create individualised reward charts in collaboration with their child, based on things their child enjoyed and using rewards that were appealing to the child. The child and parent were asked to create a list of possible rewards together
5	Effective instructions	Parents learned to use clear commands, which were short, simple and not directed as a question (e.g. can you?, would you?). This is particularly important in the context of children with cognitive difficulties, who may struggle to understand or remember long and complicated commands
6	Time out	A brief interruption of pleasant activities for the child, to act as a mild consequence. The child is removed from the situation in which the difficult behaviour occurred and is placed in a quiet and boring place, losing both attention from their parents and their freedom temporarily. As with ignoring, this is paired with praise and rewards in order to incentivise desired behaviour
7	Making a plan	Parents make a plan for times that may be challenging, such as specific locations, or specific situations. They 1. Get ready for the event—e.g. ensuring the child will be kept interested, that they are not going to be tired or hungry, and checking whether anything should be removed from the situation in advance to give the greatest chance of success, 2. Set rules with the child about their expected behaviour, 3. Set rewards for following the rules, 4. Set consequences for not following the rules, and 5. Set practice runs ahead of time
8	Relapse prevention/’looking ahead’	Parents consider what strategies have been effective for improving the difficult behaviours. They consider what to do should a new difficulty develop or an old one return. This includes monitoring the difficult behaviours, what the child and parent did and whether or not the response was successful in reducing the behaviours. They consider what may have changed, such as stopping one-to-one time. It is suggested that they use the ‘making a plan’ worksheet to prevent the problem escalating, and to set up a formal programme of rewards and consequences if this is not working

#### Session 1

##### Special Time

Goals were confirmed and the concept of one-to-one time, or ‘special time’ was discussed briefly. A time after school was identified to do the one-to-one time.

##### Monitoring

In addition, Mrs. AB was encouraged to monitor AB’s behaviour in relation to the goals throughout treatment. Specifically, she recorded the event related to the goal (positive or negative behaviour), antecedents, her response (in detail, including specific strategies discussed in the intervention), what happened afterwards and whether any strategies/responses helped in reducing the behaviours. This recording was completed in a table on a computer document that was sent to the therapist each week prior to every session and served both as a measure of patient adherence to each of the strategies and also as clinical material to review.

#### Session 2

##### Special Time

AB had not been taken home from school early for the full week. She had enjoyed the one-to-one time. After the first time, AB wanted to continue playing with her mother after the 10 min of one-to-one time had ended and her mother stayed in the room. After the third time, AB stayed in the room after the one-to-one time had finished and played on her laptop, whilst her mother did housework in the same room. AB stopped calling for her mother during the time immediately after one-to-one time. On the day of the call, Mrs. AB left the room straight after the one-to-one time and AB did not call. Afterwards she told her mother that she was happy to help her. It was discussed that in future, AB’s eagerness to help could be used to work towards the goals—for example, Mrs. AB could ask ‘are you happy to help me by playing by yourself for five minutes’.

##### Special Time with Sister

One-to-one time with AB’s sister was more difficult, as AB stood over them and interrupted. We problem solved around this with Mrs. AB. A plan was created for Mrs. AB to spend time with AB first and then her sister straight after this. The concept of immediate praise for positive behaviour was briefly discussed and Mrs. AB was encouraged to praise both AB and her sister for sitting quietly and not interrupting.

#### Session 3

##### Special Time and Praise

The first time this session was scheduled, it had to be cancelled as AB has changed medications and had been extremely sleepy and so it was difficult for Mrs. AB to implement any of the strategies. In the rescheduled session, Mrs. AB reported that although AB was asking for her as soon as she left the room, she did not do this for up to an hour immediately following the one-to-one time. In order to integrate the immediate praise, it was discussed that Mrs. AB could tell AB that she was leaving the room for one minute after the one-to-one time and ask AB not to call during this time. She could then re-enter the room and give AB specific praise for playing quietly and not calling. Mrs. AB planned to set a timer so that AB knew when the minute was up.

##### Special Time with Sister

Regarding time with AB’s sister, AB’s sister frequently asked when her turn was; it was decided that the length of time spent with each was perhaps too long and that this should be reduced in the first instance. Mrs. AB could then praise both AB and her sister for sitting quietly during each other’s one-to-one time and the time period could gradually be increased.

##### Active Ignoring

Ignoring had to be implemented carefully for AB, due to concerns about her having a seizure when no one was present. In addition, she had poor motor coordination and sometimes called her mother because she had fallen or become trapped. Mrs. AB was unsure if she could tell the difference between AB’s calls due to a genuine problem compared to her calls for attention. She was sure that she was okay when she was on the sofa, however. It was therefore decided that Mrs. AB would actively ignore repetitive question asking and any calls when AB was on the sofa.

#### Session 4

##### Active Ignoring

Mrs. AB had explained to AB that she would be ignoring repetitive questions. She ignored questions about where the cat was. After a few times, AB began to answer the questions herself. The frequency of repetitive question asking had markedly reduced. Mrs. AB had also managed to ignore times when AB was calling for attention from the sofa. As expected from behavioural principles, this resulted in escalation of the behaviours (for example, from calling to singing), but eventually the calling/singing stopped.

AB had spontaneously started to help around the house. For example, she asked Mrs. AB if she could help and had set the table during the week.

##### Special Time and Praise

The minute of leaving the room with timer and praise was going well and AB responded well to the praise. The shorter time with AB’s sister was also working well. AB allowed this to happen and did not call. AB’s sister was also reported to be happy and less frustrated; she was able to talk to Mrs. AB without AB interrupting.

AB had been deliberately annoying her sister, for example by cuddling her when she asked her not to and singing songs to her. We discussed whether AB was deliberately trying to upset her, or whether she wanted attention from her sister in the same way as her mother. Mrs. AB planned to suggest that AB’s sister may want to try one-to-one time with AB.

AB had been sent home from school due to seizures this week. We discussed that this demonstrated that it was possible for AB’s behaviour to be positive despite her physical health worsening.

#### Session 5

##### Special Time

The time that Mrs. AB spent out of the room after one-to-one time was being gradually increased and this was working well. AB’s sister had agreed to try to use the one-to-one time strategy and was happy with this. AB had not annoyed her as much in the week. AB’s mother was able to spend 30 min with her sister without being interrupted.

##### Active Ignoring

In addition to ignoring questions about the cat, Mrs. AB had also started to ignore requests for help when AB already knew how to do a task. She explained to AB that she was doing this. For example, AB would ask ‘where is ‘x’ letter on the computer?’. Mrs. AB did not answer and AB would then find it for herself. Her independence was therefore slowly increasing.

##### Effective Instructions

Effective instructions were briefly introduced.

Mrs. AB had not been called to collect AB from school all week.

#### Session 6

##### Special Time and Praise

Mrs. AB set the timer for 3 min after the one-to-one time. Although Mrs. AB was able to be out of the room for up to half an hour without AB calling, AB called her two minutes after the timer was set. We discussed that perhaps the problem was the timer and so it was agreed to remove this. Mrs AB said that there had been a 90% reduction in calling compared to the start of treatment. She had managed to spend 2 h with AB’s sister without interruption from AB. AB was frequently offering to help, for example tidying up after dinner. She again had not been sent home from school and seemed much happier.

Mrs. AB said that AB was sometimes blaming her sister for her mistakes, such as spilling drinks. This was discussed as an opportunity to promote independence (‘it doesn’t matter who spilt it but can you help by cleaning it up?’).

Due to a technical difficulty, the sheet on effective instructions had not been received and so this was resent.

#### Session 7

##### Special Time and Active Ignoring

AB’s physical health had been worse this week, with several seizures. AB had been calling Mrs. AB more frequently because she was unwell. Mrs. AB therefore spent more time with her, although she continued to ignore the repetitive question asking. It was discussed that Mrs. AB could tell AB that she could have more one-to-one time because she was unwell, thereby making her change in behaviour explicit. Removing the timer following one-to-one time had worked well and AB did not call Mrs. AB immediately after the one-to-one time. AB’s sister had continued the one-to-one time with AB. AB’s sister was encouraged to ignore any annoying behaviour and was herself rewarded when she did so.

##### Praise

Praise for independence after a mistake was working well; AB had begun to take the initiative to clean up for herself and this had in turn reduced arguments with her sister.

##### Effective Instructions

Mrs. AB had not managed to read the effective instructions sheet as AB had been so unwell.

#### Session 8

##### Special Time, Praise and Rewards

AB’s sister was working with her as a team and getting rewards, such as television time and sweets when she worked well with AB and used some of the strategies, such as one-to-one time and ignoring. Mrs. AB felt that the strategies were sustainable as they saved time in the long run due to the reduction of arguments. She was able to spontaneously spend time with AB’s sister without AB interrupting. AB was also receiving small rewards for positive behaviour and seemed happy. She was continuing to offer to help.

##### Active Ignoring

Mrs. AB felt able to distinguish times when she should ignore AB’s calling versus times when AB needed help and she should respond. She had spent twenty minutes on the phone without being interrupted by AB and immediately gave her a reward.

##### Effective Instructions

AB’s behaviour was reported to have been ‘fantastic.’ Mrs. AB had read the worksheet on effective instructions and begun to implement the advice. She was asking AB to do things in a clear and concise way and said that AB was listening and responding.

#### Session 9

##### Special Time

Mrs. AB said that AB had been spending one-to-one time with her father as well as sister. The one-to-one time with her sister continued to go well. Following one-to-one time with her father, AB had sat next to him. This had never happened before. AB’s sister was reported to be happier and her school had commented on this.

##### Active Ignoring

The ignoring was continuing to work and the repetitive question asking had stopped. AB was continuing to develop her independence, from putting in her own password for the computer to making tea and getting ready for bed by herself. Mrs. AB said that she felt okay with ending sessions as she was confident with the strategies. She had learnt that ignoring was in AB’s best interest and she was not being unkind by doing so.

#### Session 10

##### Goal Review

This was the final session prior to follow-up. Regarding the goals set at the beginning of treatment, Mrs. AB said that AB still called occasionally after one-to-one time to see if she could get her attention. However, this was much reduced, and Mrs. AB thought it might reduce further over time. Mrs. AB was able to spend time with AB’s sister when she wanted to, without interruption. On the day of the call, her sister was home from school due to illness. Previously AB would have also wanted to stay at home, but that morning she was happy to go to school. AB was continuing to develop skills of independent living, such as setting and tidying the table and getting ready for bed. Mrs. AB wanted to continue to build on these skills, with goals for the near future of AB doing her own hair, running a bath and dressing herself in the morning.

#### Follow-Up Session 1 (1 Month After Session 10)

##### Goal Review

AB had been very unwell for several weeks and during that time, Mrs. AB said that she had found the strategies and progress very hard to maintain. However, in the last few days, AB had been feeling better and Mrs. AB felt that things were getting back on track. On discussion, although AB’s behaviour had worsened somewhat whilst she was unwell, there was still progress towards the goals and the calling frequency was reduced compared to the start of treatment. Mrs. AB could still leave the room to make drinks and spend time with AB’s sister alone. In the last week, AB had spontaneously set the table and made drinks without prompting.

#### Follow-Up Session 2 (3 Months After Session 10)

##### Goal Review

Overall, progress towards the goals had been maintained and AB’s independence skills continued to increase, for example, she was now washing up. AB’s school report was reported to be the ‘best yet’ and the positive atmosphere at home continued. Mrs. AB could be self-critical and was encouraged to focus on her own strengths and to be as compassionate towards herself as she was to AB. We also discussed whether she could find a babysitter so that she could spend time without AB in the evenings.

## Results

### Session-By-Session Measures

**Fig. 1:**
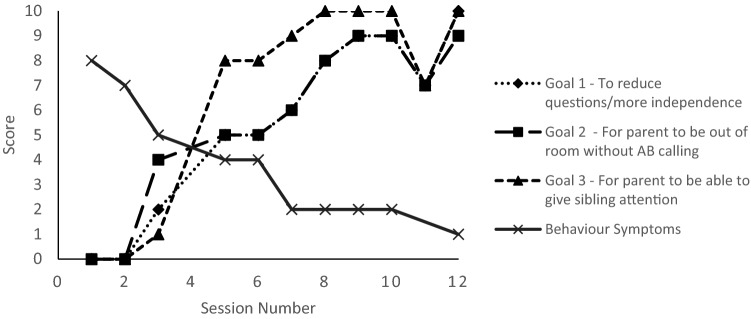
Mean scores on the session-by-session goal tracking measure for each goal and for behavioural symptoms measured by the CORC ‘How are things? ODDp’ session-by-session measure

Over the course of treatment, Mrs. AB reported that she was making progress towards the goals identified during the assessment session and by the end of treatment, she reported having fully met her goals (Fig. 1). Similarly, scores on the weekly measure of symptoms of disruptive behaviour reduced throughout (Fig. 1).

### Standardised Pre-post Measures

Scores on the SDQ, RCADS and PedsQL were variable (Table 2). All SDQ subscale scores except Conduct problems were elevated at baseline. The total score demonstrated recovery by the end of treatment but then increased at both follow-up time points. Similarly, the impact scale also demonstrated improvement by the end of treatment and then increased at follow-up. However, the emotional problems subscale and prosocial subscale demonstrated recovery at post-intervention and both follow-up time points. The only RCADS subscale demonstrating elevated symptoms at baseline was separation anxiety, which demonstrated recovery at the end of treatment but increased at follow-up. PedsQL physical subscale scores increased between baseline and post-intervention, indicating improved Quality of Life. However, psychosocial functioning scores decreased at post-intervention. All scores increased at the first follow-up time point and then decreased at the second follow-up.

**Table 2:** Scores on standardised measures at baseline, end of treatment and follow-up

Scale	Subscale	Time point			
		Baseline	End of treatment	Follow-up 1	Follow-up 2
SDQ	Total (range)	21 (very high)	11 (close to average)	17 (high)	18 (high)
	Emotional problems (range)	6 (high)	2 (close to average)	3 (close to average)	3 (close to average)
	Conduct problems (range)	1 (close to average)	0 (close to average)	0 (close to average)	0 (close to average)
	Hyperactivity (range)	7 (slightly raised)	6 (slightly raised)	7 (slightly raised)	8 (high)
	Peer problems (range)	7 (very high)	3 (slightly raised)	7 (very high)	7 (very high)
	Prosocial (range)	6 (low)	9 (close to average)	9 (close to average)	8 (close to average) recovered
	Impact (range)	7 (very high)	3 (very high)	6 (very high)	7 (very high)
RCADS T-score	Total (T-score)	48 (68)	18 (37)	31 (45)	39 (51)
	Social phobia (T-score)	10 (52)	5 (35)	7 (39)	10 (45)
	Panic disorder (T-score)	7 (74)	0 (36)	2 (43)	4 (49)
	Separation anxiety (T-score)	12 (> 80)	3 (53)	11 (> 80)*	13 (> 80)
	Generalised anxiety (T-score)	6 (58)	1 (32)	1 (32)	1 (32)
	Obsessive-compulsive disorder (T-score)	0 (43)	0 (35)	0 (35)	0 (35)
	Major depression (T-score)	13 (78)	9 (54)	11 (59)	11 (59)
PedsQL	Physical functioning	34.375	43.75	40.625	31.25
	Emotional functioning	60	45	95	80
	Social functioning	5	15	15	10
	School functioning	15	35	30	30
	Psychosocial	80	31.67	46.67	40

^*^One item (“my child feels scared to sleep on his/her own”) was marked as N/A as Mrs AB said that she slept with AB every night due to her seizures

### Diagnostic Measure

AB did not meet threshold for a mental health disorder before or after intervention according to the clinician rated DAWBA.

### Experience of Service and Qualitative Interview

On the CHI-ESQ, Mrs. AB said that she ‘was given simple easy to follow advice that was practical and had positive responses from my daughter very quickly’ and that she received ‘excellent service which has enabled me to give more time to one daughter and has improved the other daughter’s behaviour immensely’. Mrs. AB was also interviewed as part of a related qualitative study (Bennett et al., 2018). In her own words, the intervention.

can make changes to not only me but to the actual person who has epilepsy and make your life so much better, and make her whole family life better. I never thought we’d get any help, and that help…it doesn’t have to take a long time, it can be ten phone calls, it doesn’t have to be intense, it’s something that’s simple, that as long as you do it, it can have a huge impact on the family’s life. I think when you’re dealing with something like epilepsy, that’s hard enough to deal with and when you have behavioural problems on top of that it really restricts anything you can do in your life and even going out and having any family life, the rest of the family, so if you can even get a little bit of help and know how to deal with certain situations, it can have a huge impact even to the point where it can keep families together.

She reported that the main thing she had learnt was that small changes could make a big impact on AB’s behaviour and that the intervention gave her the confidence to make those changes.

## Discussion

The results of this case report suggest that a 10-week guided self-help teletherapy intervention for behavioural difficulties was helpful in reducing specific aspects of problematic behaviour, including those associated with separation anxiety in a young person with epilepsy and additional complex needs. Changes in weekly measures, in addition to qualitative feedback from the young person’s parent, suggests that the strategies were effective for working towards specific goals and reducing symptoms of disruptive behaviour, despite her declining physical health. Mrs. AB completed all sessions of therapy, was able to complete the worksheets and reported that she implemented the interventions and found them helpful, suggesting that the intervention is feasible and acceptable to parents. The family was highly motivated and dedicated to the programme and it is possible and likely that other families would require more intensive support and input than guided self-help, but the case demonstrates that this relatively low-level standard evidence-based intervention may be sufficient for some families. Although it was delivered in the context of a stepped-care service, in which onward referral for more intensive treatment would have been possible, such referral was not needed in this case.

The variability in outcome on standardised measures is a key aspect to consider for future studies investigating interventions for these types of complex presentations of both physical and mental health difficulties. Whilst the total SDQ score and, importantly, impact scales did not demonstrate sustained improvement at follow-up, relevant subscales that directly related to the goals of treatment (emotional problems and prosocial) did. Unsurprisingly, subscales relating to developmental difficulties, not designed to be ‘treated’ by this intervention alone, such as ADHD and autism (i.e. hyperactivity, peer problems) did not. Similarly, whilst goal progress demonstrated clear improvement in symptoms of separation anxiety that were sustained at follow-up, the RCADS separation anxiety subscale did not. Again, this score needs to be considered in light of the presentation of the child; the parent felt that one of the items was not applicable because she wanted the child to share her bed because of seizures. This example demonstrates some of the complexities around using such standardised measures, particularly those that are not normed for a population with intellectual disabilities. This may also partly explain the lack of significant change in the impairment score. The impairment questions ask whether the difficulties upset or distress the child and whether they interfere with home life, friendships, classroom learning and leisure activities. It may therefore be difficult to distinguish impairment related to mental health from impairment related to physical health. There is further complication from the variable seizure pattern; AB’s physical health deteriorated through the treatment and therefore it is understandable that she and her mother may have been more intensely attached to each other during this time period. It therefore may be that individualised goal-based measures are more appropriate in this population. In addition, tracking wider outcomes in other areas, such as absence from school, which was noted to decrease in this case, is helpful.

The main strength of this study is that it was undertaken in routine clinical practice, exclusion criteria for the study were minimal and the complexity seen in AB, including social communication difficulties and intellectual disability, is typical of the families seen within paediatric neurology (Reilly et al., 2014). There were a number of limitations; the uncontrolled single case does not allow us to confidently attribute any change to the intervention, particularly as we did not collect multiple baseline data-points. It is impossible to rule-out whether changes may have occurred because of external, unrelated factors or whether the impact was affected by changes in AB’s epilepsy course. We also did not use a formal measure of adverse events which may have provided additional evidence as to whether such interventions are safe for children with epilepsy. Adverse effects in psychotherapy include deterioration of symptoms, new symptoms, stress, stigma, dependence on therapist and hopelessness if therapy does not improve symptoms (Rozental et al., 2016). An important potential negative effect of parenting interventions for behavioural difficulties in children is an ‘extinction burst’ in response to active ignoring. As the child is no longer reinforced for previously reinforced behaviour, they are likely to increase the difficult behaviours in the short term in order to gain parental attention. With continued ignoring, the behaviours should decrease in accordance with behavioural principles of punishment and reinforcement. However, if the parent responds to these more difficult behaviours, this reinforces them, meaning the behaviour may worsen in the long-term. Given the pressures on families of children with chronic illness, it is important that families who undertake such intervention know of the work involved and potential risks. Clinicians also need to ensure that families are adequately supported through the initial extinction burst.

This case provides initial evidence that it may be possible to use a brief, cost-effective guided self-help teletherapy parenting intervention in the context of behavioural difficulties in children and young people with epilepsy. Such intervention is likely to only be a component of a broader intervention, which may be needed to improve more general impairment. The detail provided here will allow other clinical services to replicate similar approaches. Future research is needed to investigate the effectiveness of this intervention, with a larger sample and a control group.
